# Gene expression related to trehalose metabolism and its effect on *Volvariella volvacea* under low temperature stress

**DOI:** 10.1038/s41598-018-29116-z

**Published:** 2018-07-20

**Authors:** Xu Zhao, Xiaoxia Song, Yapeng Li, Changxia Yu, Yan Zhao, Ming Gong, Xuexiang Shen, Mingjie Chen

**Affiliations:** 1 Institute of Edible Fungi, Shanghai Academy of Agricultural Sciences, Shanghai, 201403 P.R. China; 2 National Engineering Research Center of Edible Fungi, Key Laboratory of Edible Fungi Resources and Utilization (South), Ministry of Agriculture, Shanghai, 201403 P.R. China

## Abstract

The mechanism of the low temperature autolysis of *Volvariella volvacea* (*V*. *volvacea*) has not been thoroughly explained, and trehalose is one of the most important osmolytes in the resistance of fungi to adversity. The present study used the low temperature sensitive *V*. *volvacea* strain V23 and the low temperature tolerant strain VH3 as test materials. Intracellular trehalose contents under low temperature stress in the two strains were measured by high performance liquid chromatography (HPLC). Quantitative real-time PCR (qPCR) analysis was carried out to study the transcriptional expression differences of enzymes related to trehalose metabolism. And trehalose solution was exogenously added during the cultivation of fruit bodies of *V*. *volvacea*. The effect of exogenous trehalose solution on the anti-hypothermia of fruit bodies was studied by evaluating the sensory changes under low temperature storage after harvest. The results showed that the intracellular trehalose content in VH3 was higher than that in V23 under low temperature stress. In the first 2 h of low temperature stress, the expression of trehalose-6-phosphate phosphatase (*TPP*) gene involved in trehalose synthesis decreased, while the expression of trehalose phosphorylase (*TP*) gene increased. The expression of *TPP* gene was almost unchanged in VH3, but it decreased dramatically in V23 at 4 h of low temperature stress. The expression levels of *TPP* and *TP* genes in VH3 was significantly higher than that in V23 from 6 h to 8 h of low temperature stress. *TP* gene may be a crucial gene of trehalose metabolism, which was more inclined to synthesize trehalose during low temperature stress. In addition, the sensory traits of *V*. *volvacea* fruit bodies stored at 4 °C were significantly improved by the application of exogenous trehalose compared with the controls. Thus, trehalose could help *V*. *volvacea* in response to low temperature stress and high content of it may be one of the reasons that why VH3 strain was more tolerant to the low temperature stress than V23 strain.

## Introduction

Biological growth, reproduction, behavior, quantity and distribution are affected by temperature. Under low-temperature stimulation above zero, the fungus is under environmental osmotic stress. The physiological metabolism of the cells will lead to a series of stress responses to resist the osmotic stimulation of low temperature stress. Cells can prevent the outflow of intracellular water by promoting the accumulation of one or more specific solutes, maintaining osmotic pressure and ensuring the normal functioning of various physiological functions^1^. According to their characteristics, these particular solutes can be divided into two categories: inorganic ions, such as K^+^ and Na^+^, from the outside environment and organic solutes synthesized in the cell, including polyols and nitrogen compounds, such as soluble sugars (e.g., trehalose and sucrose), amino acids and their derivatives (e.g., proline and betaine)^2,3^. As an important osmotic adjustment substance, soluble sugar can increase the concentration of cytoplasm, reduce the freezing point and relieve the excessive dehydration of cytoplasm^4^. Trehalose, an important nonreducing disaccharide in soluble sugars, has a maximum content of more than 20% of the dry weight of organisms in fungi such as Saccharomycetes and Basidiomycetes^5^.

Trehalose is the most stable sugar in natural disaccharides^6^ and is widespread in organisms. This sugar was initially isolated in 1832 by WIGGERS in Claviceps purpurea from *Secale cereale* L.^7,8^. Under normal physiological conditions, the content of trehalose in the fungus is in a stable state, but the intracellular trehalose content typically changes when the cells are under osmotic stress^9^. The trehalose in fungi can be considered as a carbon storage source to provide energy after decomposing into glucose at spores germination or fruit body stages. This sugar can also be used as an effective cytoprotective agent under extreme temperature, radiation and dehydration environment^10^. However, the study of endogenous trehalose in edible fungus against the effect of low temperature stress is rare, and in the reported yeast studies, the higher levels of intracellular trehalose yeast strain often have a high tolerance of adversity caused by hypertonic conditions^11,12^. Trehalose is considered to be a natural protective agent in yeast^13^. In addition to yeast, there are also several exploratory studies using exogenous trehalose in the preservation of edible fungus strains, such as *V*. *volvacea* and *Lentinula edodes*, at low temperatures. However, exogenously added trehalose can also improve the frost resistance of edible fungi cells, but this effect warrants further verification.

The mushroom *V*. *volvacea* (Bull.ex.Fr.) Sing is an edible fungus with a distinctive savory flavor and high nutrient content, which is internationally recognized as “a very good protein source”^14,15^. Because *V*. *volvacea* is a high-temperature mushroom species, it is sensitive to low temperature. Under the conventional storage temperature of 4 °C, wilting, effluent or even decay phenomena occur, which limits the growth of the *V*. *volvacea* industry. Although there have been studies on the low-temperature autolysis of *V*. *volvacea*, its specific mechanism of action has not been clearly explained. Therefore, by reference to the amino acid sequence of trehalose metabolism-related enzymes in homology in other edibe fungus, the gene sequences of these enzymes were screened from the whole genome of *V*. *volvacea* by blast. Moreover, the metabolic pathway of trehalose in the *V*. *volvacea* was inferred, and the gene expression of the related enzymes was quantitatively analyzed. The content of intracellular trehalose in different treatments was determined. Furthermore, trehalose solution was exogenously added during the *V*. *volvacea* cultivation process, and fruit bodies were examined to explore the tolerance to low temperature. This study will provide scientific research ideas on the effect of trehalose during low temperature stress and a theoretical basis, however, the genetic mechanism of *V*. *volvacea* autolysis under low temperature, as well as genetic transformation of functional genes need to be further studied.

## Materials and Methods

### Test strains and collection of *V*. *volvacea* mycelia

V23 strain used in the present study, is a low temperature-sensitive strain. The VH3 strain was derived from V23 by mutagenesis, which is more tolerant to low temperature than V23. The two strains were supplied by the Institute of Edible Fungi, Shanghai Academy of Agricultural Sciences.

The V23 and VH3 strains were introduced into a solid medium and incubated at a constant temperature of 32 °C for 4 days. The strains were later transferred to a flask containing liquid medium and incubated at 150 rpm for 5 days at 32 °C.

After hypha culture, the flask was transferred to ice bath at 0 °C for 2, 4, 6 and 8 h. The blank control group was not transferred to ice bath. Sterile-filtered mycelia were rinsed several times with sterile water, blotted dry on sterile absorbent paper, collected in a sterile centrifuge tube, frozen in liquid nitrogen, and subsequently stored at −80 °C.

### Trehalose extraction and quantitative determination

To determine the trehalose content, the mycelia were freeze-dried and ground to powder using a TissueLyser. 1 g of dry powder from *V*. *volvacea* mycelia was extracted by ultrasonic water bath in 100 mL distilled water for 1 h. The extracted solution was centrifuged at 13400 g for 10 min. The supernatant was filtered through a membrane filter (0.45 μm). Then a high performance liquid chromatography (HPLC) was developed for the determination of trehalose. By use of SUGAR SC1011 column (300 mm × 8 mm) as the chromatographic column and purified water as mobile phase, trehalose was separated completely at the refractive index detector with the flow rate of 0.5 mL/min at the temperature of 65 °C. The injection volume was 10 μL.

### Extraction and reverse transcription RNA of *V*. *volvacea* mycelium

Total RNA from *V*. *volvacea* mycelia was extracted by using Trizol (TIANGEN, China) according to the manufacturer’s instructions. The RNA was dissolved in 50 μL water (DEPC pretreatment) and detected by 1% agarose gel electrophoresis. After removal of the genomic DNA (Table S1), the RNA was reverse transcribed into cDNA according to the instructions of the reverse transcription kit (PrimeScript RT reagent Kit with gDNA Eraser), used as a template for the fluorescence quantitative real-time PCR (qPCR) reaction, and stored at −20 °C until further use (Table S2).

### Obtaining the target gene coding region, design and synthesis of qPCR primers

According to the accession number of the genes related to trehalose metabolism in the homologous species, the sequence of the coding region of each gene was obtained from NCBI, and the complete genome sequence information of *V*. *volvacea* (https://www.ncbi.nlm.nih.gov/) was aligned. In GenBank, blastx was used to compare the DNA sequence and eukaryotic protein to remove introns (GT-AG). The potential introns were removed to obtain the coding region of the reference and target genes. According to the stability and high copy number of the internal standard genes of *V*. *volvacea* strains under low temperature, tubulin alpha (*TUB*) was used as an internal standard to quantify the target gene according to prevous study^16^. Primer 5.0 software was used to design primers based on the nucleotide sequence of the coding region of genes involved in trehalose metabolism. The length of the primers was typically between 18 and 25 bp.

### Amplification of the target fragment and the preparation of standard plasmid

The target fragment was amplified by conventional PCR (Table S3) and the product was examined by 1% agarose electrophoresis. The purified PCR product was ligated into the pGEM^®^-T vector to construct a standard plasmid to generate a standard curve for qPCR^17^ (Table S4).

### Fluorescent quantitation of genes

The cDNA of *V*. *volvacea* mycelia treated at 0 °C for different time was used as the template, and the quantitative amplification of the target gene and internal standard gene was performed. Three replicates were set for each sample, and ddH_2_O was used as a blank control (amplification reaction system shown in Table 1). qPCR was performed using the ΔΔCT method.

**Table 1:** Reaction system of qPCR.

Reagent	Volume
SYBR Premix Ex Taq^TM^ (2×)	10 μL
Forward primer (10 μmo1/L)	0.4 μL
Reverse primer (10 μmo1/L)	0.4 μL
Rox	0.4 μL
ddH_2_O	6.8 μL
cDNA	2 μL
Total	20 μL

The reaction conditions: 95 °C 20 s; 95 °C 5 s, 60 °C 15 s, 72 °C 15 s, total 40 cycles. The reaction fluid was prepared in an ice bath.

### Exogenous trehalose spray treatment

Next, 95% cottonseed hulls and 5% lime were weighed according to the mass ratio, and the pH value was 10. After loading and inoculation, the culture materials were used for the fruit test.

Trehalose treatment group (T): spray 10% trehalose solution; each basket sprayed with approximately 100 mL. After 6 days of vaccination, water was added once. Each basket of water added was approximately 120 mL.

Blank control group (CK): spray water; each basket sprayed with approximately 100 mL. After 6 days of vaccination, water was added once. Each basket of water added was approximately 120 mL.

### Storage after harvest

*V. volvacea* is in the egg shape, and the membrane is not cracked prior to harvesting. The fruit body was picked up and stored in a plastic tissue culture flask with a diameter of 9 cm and a height of 8 cm. The kraft paper was sealed and stored at 4 °C. Under a humidity of 80% for 0, 6, 12, 18, 24, 30, 36, 42, and 48 h.

### Index determination and data analysis

#### Sensory index determination

Sensory index: sensory evaluation using the digital scoring method, according to the indicators listed in Table 2. The sum of the scores of each index is the final score.

**Table 2:** Sensory evaluation criteria.

Sensory index	Scores			
	4	3	2	1
Hardness	Texture is full	Slightly inelastic	Soft texture	Soft texture
	Hard	Hardness is acceptable		Not edible
Flavor	Odorless	Fresh mushroom flavor	Slightly fresh	No fresh mushroom flavors
	Typical mushroom flavor	Slight odor	Mushrooms have obvious smell	Serious odor
		Market is acceptable	Limited application	Not edible
Color	No change	Small part of browning	Most browning	Whole mushroom browning
Effluent condition	None	Slight water	Water	Large amount of water
	The surface is not sticky	The surface slightly sticky	The surface is sticky	The surface is very sticky

#### Weight loss rate determination

Weight loss rate = (fresh weight before storage-fresh weight after storage)/fresh weight before storage × 100%.

#### Morphometric determination

To measure the changes in the morphological trend of the fruit bodies during storage, the diameter of the bottom is defined as the bottom diameter in the erect state of the conical fruit body. The diameter of the boundary between brown and white in the middle of the fruit body is defined as the middle diameter. Each index measurement was obtained using vernier calipers. Each treatment group was selected after the determination of the average of 10 fruit bodies for statistics.

Bottom diameter reduction = (initial bottom diameter-post-storage bottom diameter)/initial bottom diameter × 100%.

Reduced middle diameter = (initial middle diameter-middle diameter after storage)/initial bottom diameter × 100%.

#### Data analysis of differences

All extracts and determinations were performed in 3 replicates, and the data were analyzed using SPSS (Version 11.0) software with an analysis difference at *P* = 0.05.

### Data availablity

The dataset supporting the conclusions of this study is available and we have agreed to share the dataset. You can contact us by email to obtain the raw data in our manuscript.

## Results

### Changes of trehalose content in *V*. *volvacea* mycelia subjected to low temperature treatments at different times

**Figure 1:**
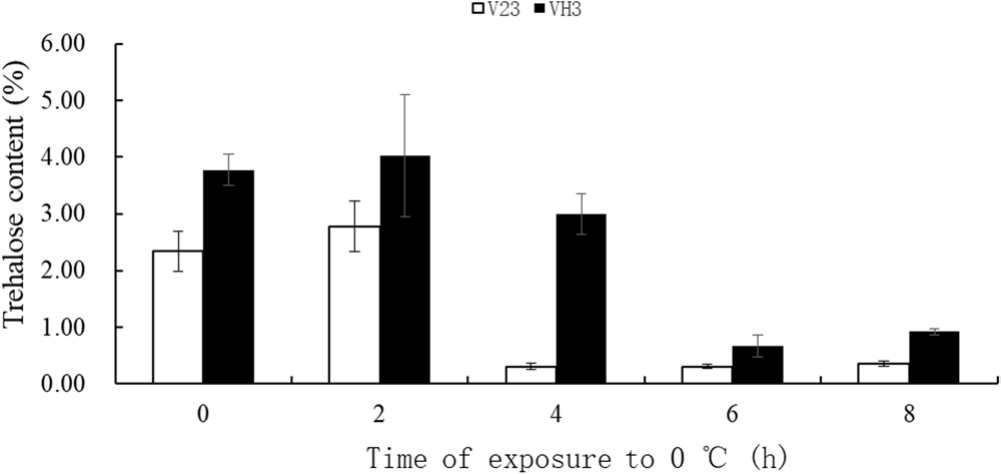
Changes in the trehalose content in mycelia of *V*. *volvacea* at 0 °C for 0, 2, 4, 6 and 8 h. The data are provided as the means ± SD from three independent replicates (n = 3).

The mycelia of *V*. *volvacea* exhibited optimal growth at 28–32 °C. Trehalose has been reported to play an important role in sustaining the growth of microorganisms under temperature stress^18^. However, little is known regarding the role of trehalose in the response to low temperature stress in *V*. *volvacea*. Therefore, we messured the contents of trehalose during low temperature stress by HPLC. During exposure to low temperature, the trehalose content in V23 and VH3 kept unchanged at the first 2 h compared with their respective control (0 h). However, the trehalose content in both strains decreased with storage time, especially decreased dramatically in V23 at 4 h of low temperature stress (Fig. 1). Under the same time of low temperature stress, the trehalose content in VH3 strain was significantly higher than that in V23 strain.

### Changes of genes expression related to trehalose metabolism

**Figure 2:**
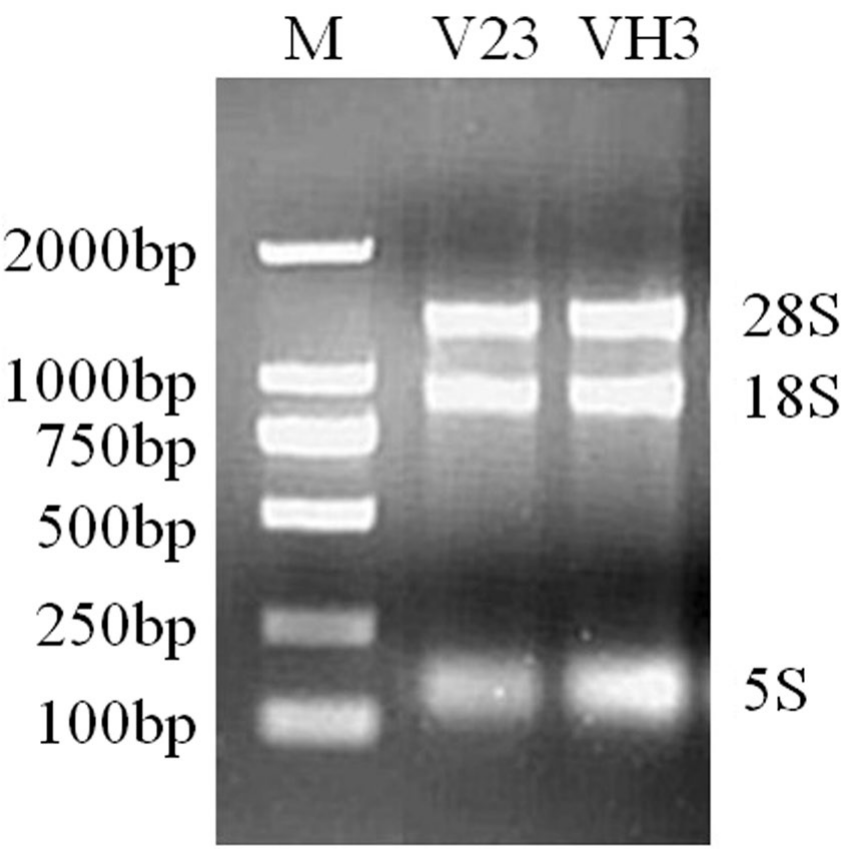
Electrophoresis of the total RNA of *V*. *volvacea* mycelia. The extracted total RNA was intact, the strip was clear, 28S was twice as bright as 18S, and the 5S dispersion was not obvious, indicating that the RNA was not degraded. Figure was edited as described in methods, for original figure referred to Figure S1.

**Figure 3:**
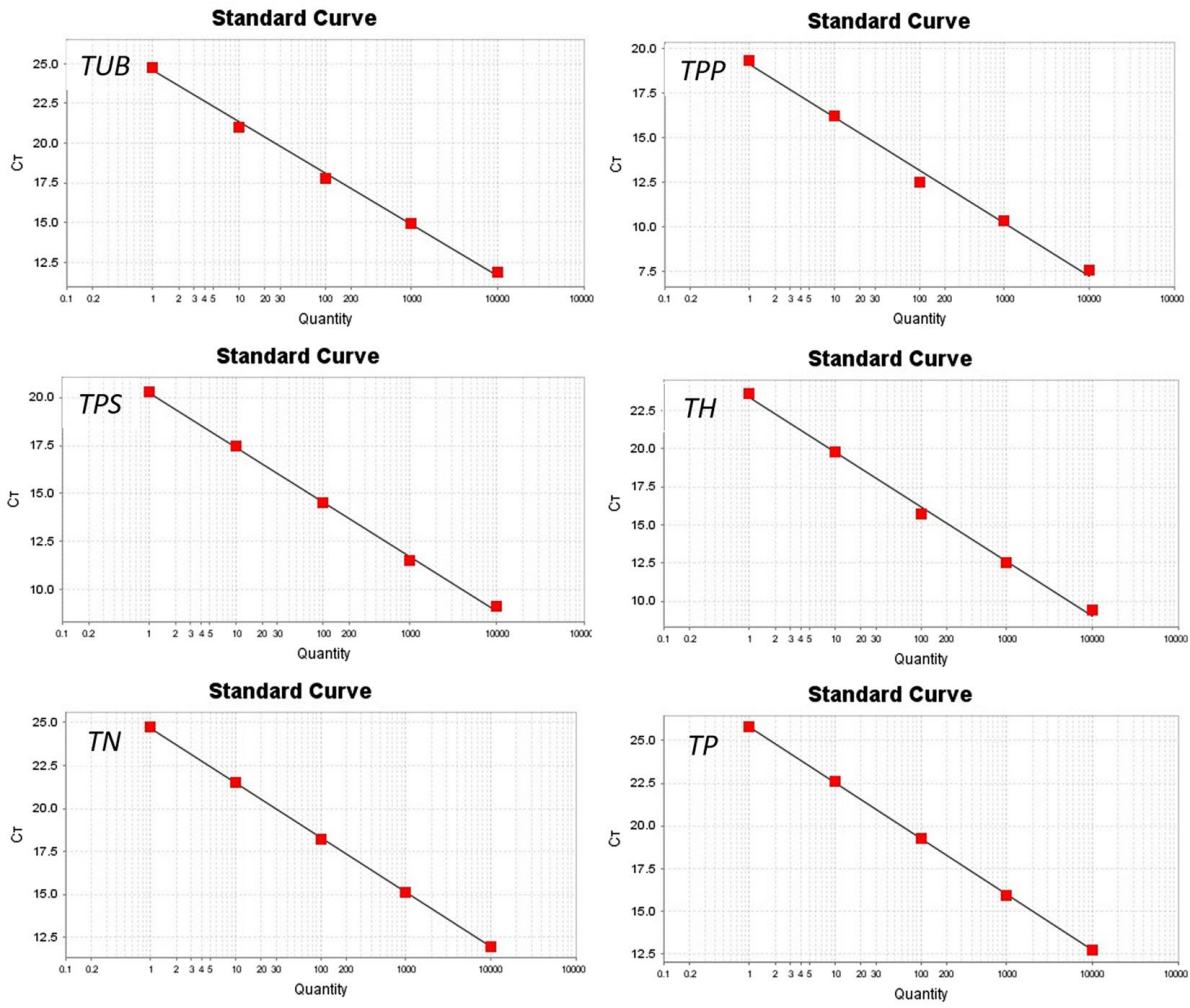
Standard curves of the target genes. The standard curve can be used as an accurate quantification of unknown samples. The standard curve R^2^ of the target gene was greater than 0.99, the slope K value satisfies |ΔK| ≤ 0.1 (Table S6). qRT-PCR was therefore performed using the ΔΔCT method. Biology duplications are three times.

**Figure 4:**
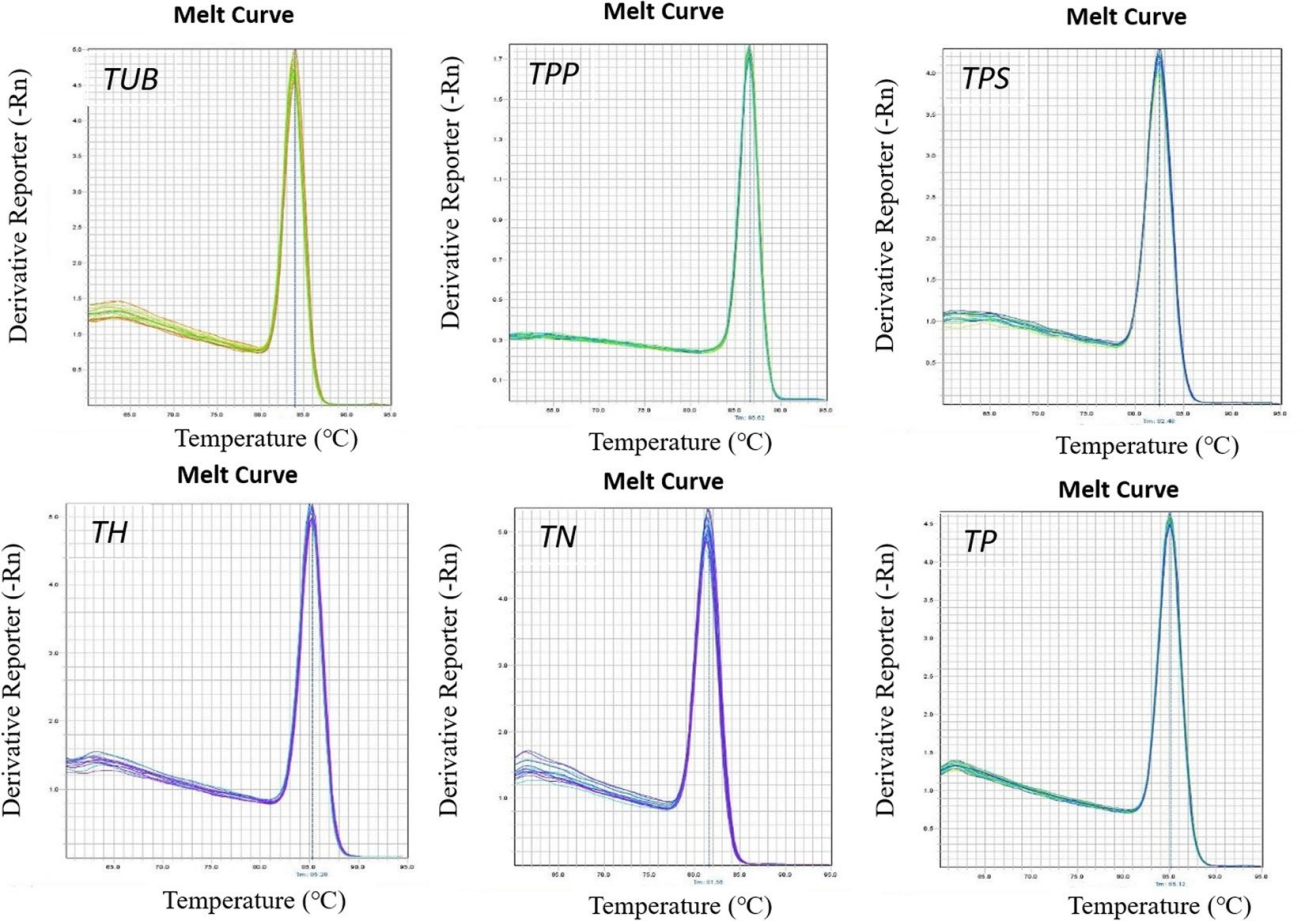
Melting curves of the target genes and the internal standard gene. The single peak of the melting curve of the target gene and the internal standard gene indicated that the specificity of the amplification was strong and that the result was feasible.

The quality of total RNA extraction directly affects the quality of template cDNA and the accuracy of quantitative PCR results. The quality of extracted total RNA electrophoresis test is shown in Fig. 2, revealing an intact, clear band, indicating that the RNA has not been degraded. After routine PCR amplification, the band of the target gene was consistent with the length of the primer-specific fragment (Table 3), and a single band was detected for each gene, indicating that the primer has good specificity and can be used for qPCR amplification. Cloning and sequencing results (Table 4) showed that the amplified target gene fragment sequence and the original sequence were precisely the same. The standard curve R^2^ of the target gene was greater than 0.99, indicating a good linear relationship (Fig. 3 and Table S5). The standard curve can be used as an accurate quantification of unknown samples. The single peak of the melting curve of the target gene and the internal standard gene (Fig. 4) indicated that the specificity of the amplification was strong and that the result was feasible.

**Table 3:** Primers used for qPCR.

Primer	Base number (bp)	Gene sequence
*TPS-F*	20	5′-GATTACCACCTGTTACTTGC-3′
*TPS-R*	19	5′-GACGAGCGTAGGAGTATGT-3′
*TPP-F*	21	5′-GATCTGGAAACGGGATCTGTC-3′
*TPP-R*	21	5′-CCCACTCAACAACCAAACCTC-3′
*TH-F*	21	5′-GGCGAAGAAGGAAACACACAC-3′
*TH-R*	25	5′-CGTAGCATTACCACCATTAACAACA-3′
*TN-F*	24	5′-GTATTCCGTTTATTGTGCCTGGTG-3′
*TN-R*	20	5′-TATGAGCGACTGCCGTTGAG-3′
*TP-F*	20	5′-TCAGGTTCCTCCGTGCTCTT-3′
*TP-R*	22	5′-TTGTGGTTGTTCTTGGTGGTTC-3′

**Table 4:** Cloning and sequencing results.

Gene	Cloning and sequencing results	Sequence length (bp)
*TUB*	CCAACACTACCGCTATCTCCTCGGCTTGGAGTCGCCTTGATCACAAGTTCGACCTCCTCTATTCGAAGCGTGCTTTCGTGCATTGGTACGTTGGTGAGGGTATGGAGGAAGGTGAA	116
*TPP*	GATTACCACCTGTTACTTGCCCCGAGGATGATCCGTGAACTCATCCCTGAAGCTGTCCTCGGCTTGTTCGTGCACACACCATTCCCAAGCAGCGAAGTTTTCCGATGCCTACCTCGCCGCAAGGAGATACTCGATGGCATGCTAGGCGCCAACCTTGTTTGCTTCCAAACATACTCCTACGCTCGTC	187
*TPS*	GATCTGGAAACGGGATCTGTCGAGGCCGTTGGTCAATGATACGACCGGGCTGCCGCTGGAGGTGTTGGAGGATCTGAAAAAGTTGGCGGAGGATGAGAGGAGGAATGAGGTTTGGTTGTTGAGTGGG	127
*TH*	GGCGAAGAAGGAAACACACACAATGCAACCCTCACCGGACCTGAAGCTGATATCAACACTTTGGAAGGCACTGTTGTTAATGGTGGTAATGCTACG	96
*TN*	GTATTCCGTTTATTGTGCCTGGTGCCCGCTTCAATGAGCTTTACAACTGGGATTCCTACTTCATCTCGTTGGGCCTGCTGGTCGACGGACAAGTGAGCATGGCCAAAGGCATGGTCGACCACTTCATCTTTGAAATCAAACACTACGGCAAAATCCTCAACGGCAGTCGCTCATA	175
*TP*	TCAGGTTCCTCCGTGCTCTTGACGTTAATGCAGCTTGGTATGTGCCAAACCCATCACCAAGCGTCTTTCGAACCACCAAGAACAACCACAA	91

**Figure 5:**
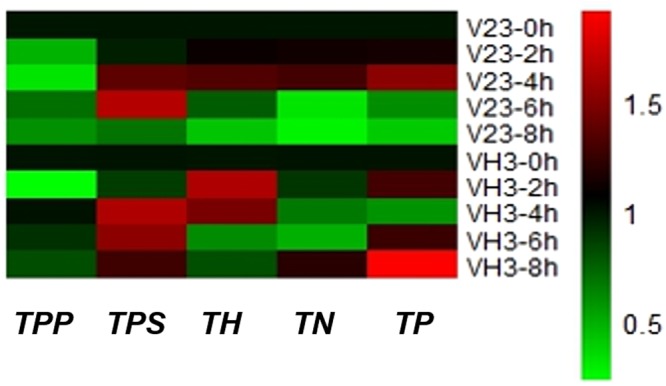
Overall changes in the expression of each gene in the trehalose metabolic pathway under low temperature stress. Red means up-regulation, green means down-regulation.

**Figure 6:**
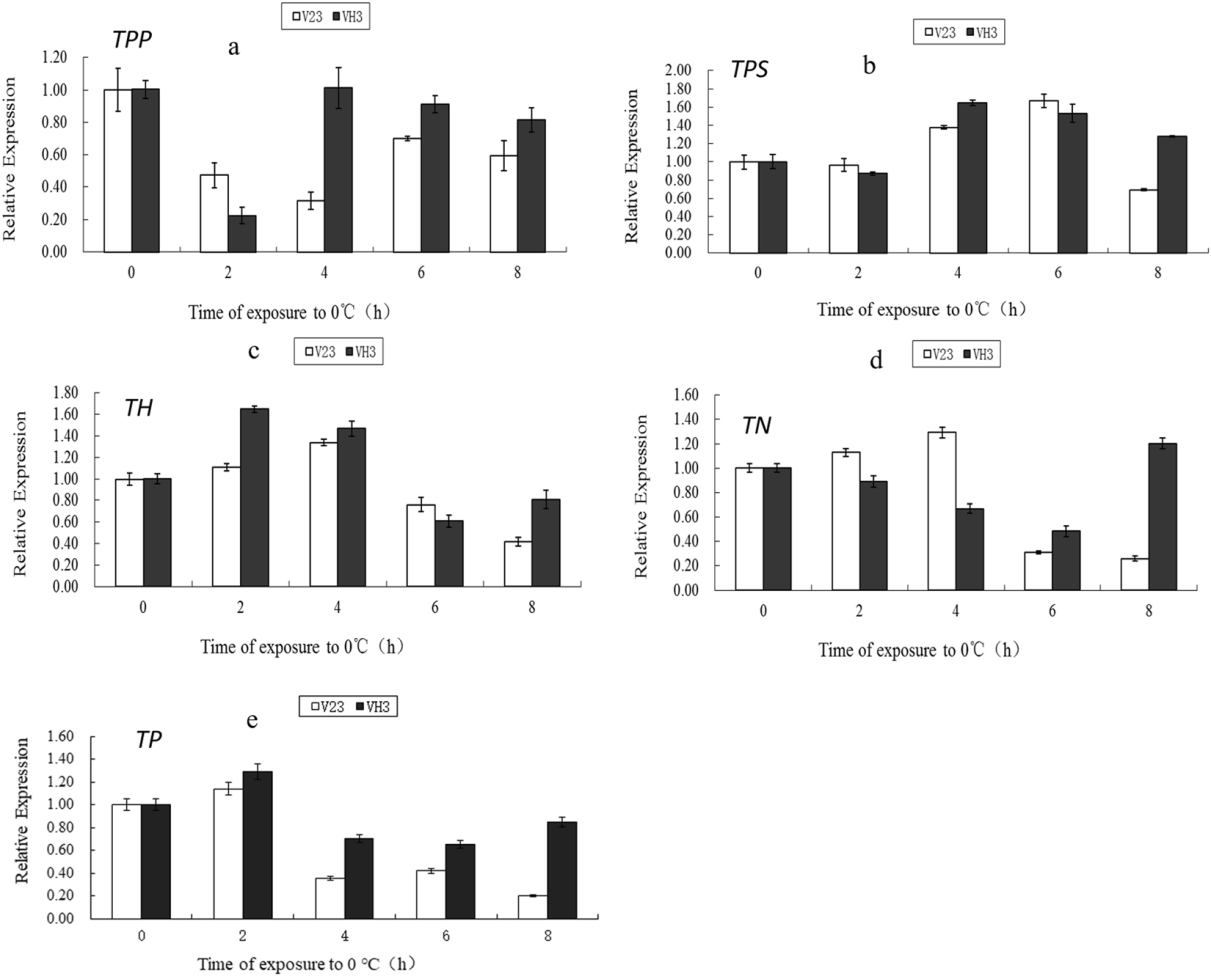
Relative expression of each gene in the trehalose metabolic pathway of strains V23 and VH3. Values are means ± standard errors (n = 3). The letters in the picture represent expression of each gene, i.e. (**a**) *TPP*; (**b**) *TPS*; (**c**) *TH*; (**d**) *TN*; (**e**) *TP*.

The relative expression of each gene in the trehalose metabolic pathway was determined for strains V23 and VH3. The results (Fig. 5) showed that the expression of *TPP* gene (Fig. 6a and Table S7) in strain V23 decreased significantly after low temperature treatment for 2 h. The gene expression decreased with prolonged low temperature treatment. However, the expression of *TPP* gene increased at 6 h but it was still lower than that at 0 h. At low temperature for 8 h, the gene expression also decreased. During the entire low temperature treatment, the expression level of *TPP* gene in V23 strain was lower than that in the 0 h untreated strain. The *TPP* gene of *V*. *volvacea* strain VH3 decreased significantly after 2 h at low temperature. However, the gene expression significantly increased after cold treatment for 4 h. With the extension of the low temperature treatment time, *TPP* gene expression showed a slight downward trend. As shown in Fig. 6b and Table S8, the expression level of trehalose-6-phosphate synthase (*TPS*) gene of the V23 strain of *V*. *volvacea* slightly decreased at 2 h compared with the 0 h. With the extension of low temperature treatment, the gene expression increased. At low temperature for 8 h, *TPS* gene expression reduced significantly. The expression level of *TPS* gene of strain VH3 decreased at 2 h of low temperature treatment. With the extension of low temperature treatment, the gene expression increased at 4 h or 6 h. At low temperature for 8 h, *TPS* gene expression decreased but it was still higher than that at 0 h. As shown in Fig. 6c and Table S9, the trehalase (*TH*) gene expression of the V23 strain of *V*. *volvacea* slightly increased at 2 h. The gene expression increased with prolonged cold treatment at 4 h. However, the *TH* gene expression decreased at 6 and 8 h. The *TH* gene of *V*. *volvacea* strain VH3 was significantly increased after 2 h at low temperature. The gene expression was reduced at 4 h but it was still higher than that at 0 h. With the extension of low temperature treatment, *TH* gene expression decreased at 6 h or 8 h. As shown in Fig. 6d and Table S10, the alpha-trehalose-neutral trehalase (*TN*) gene expression in the V23 strain of *V*. *volvacea* increased after 2 and 4 h compared to that at 0 h and later decreased with the prolongation of low temperature treatment. With the delay of low temperature, the *TN* gene in *V*. *volvacea* strain VH3 showed a decreasing trend, and the expression level at 6 h decreased to 0.48 times of that detected at 0 h. As shown in Fig. 6e and Table S11, the expression of trehalose phosphorylase (*TP*) gene was highest at 2 h both in V23 strain and VH3 strain. Compared with their respective control (0 h), the expression of *TP* gene had a significant decline after 2 h of low temperature stress, however, the *TP* gene expression in VH3 strain was significantly higher than that in V23 strain during 4–8 h of low temperature stress.

### Effect of exogenous trehalose on the low temperature tolerance of *V*. *volvacea* fruit bodies

#### Sensory

**Figure 7:**
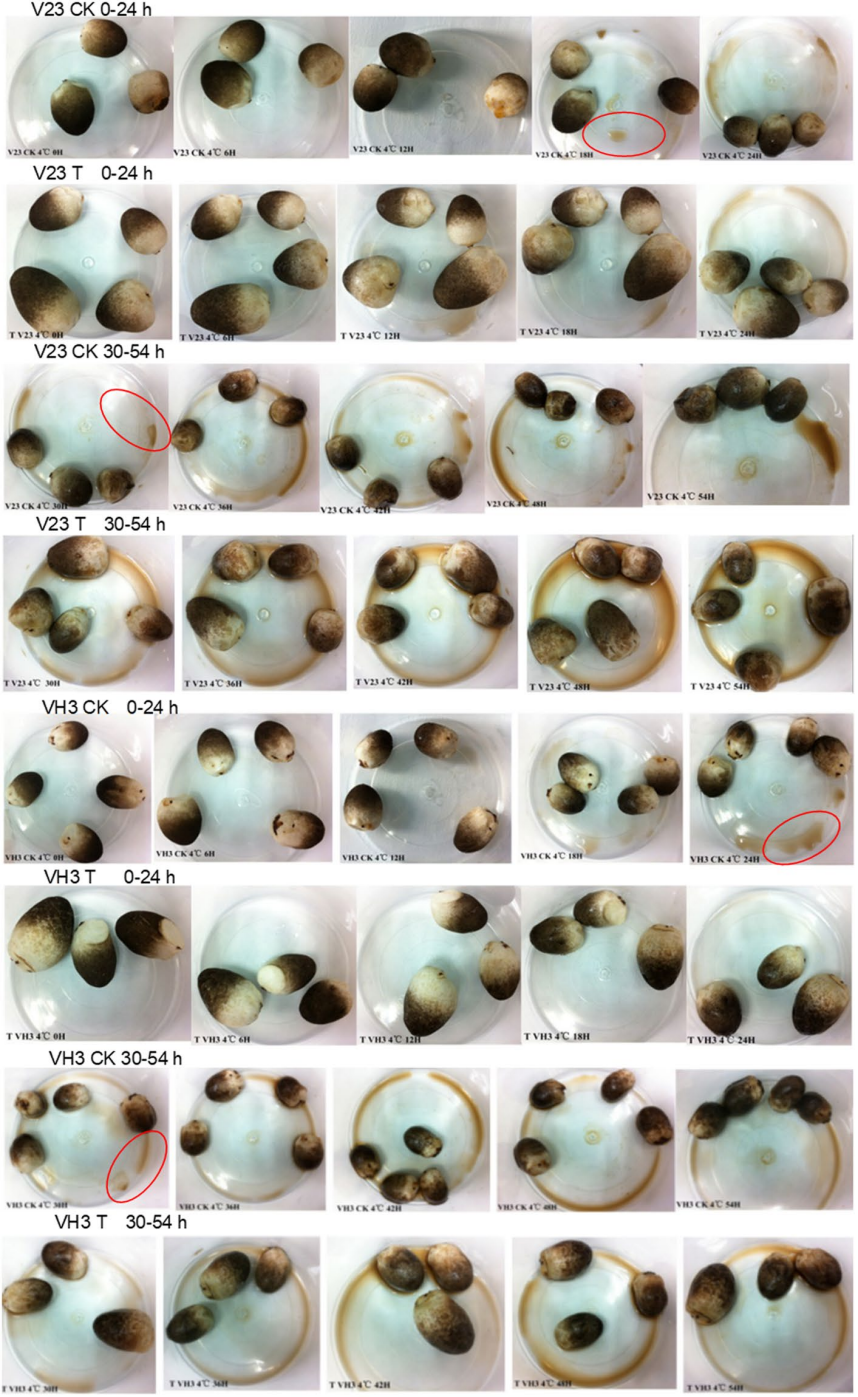
Sensory quality of the *V*. *volvacea* fruit bodies under different treatment conditions at 4 °C. Every six hours, we took a picture of the sensory images of the fruit bodies and observed the morphological changes of V23 and VH3 at 4 °C. Among them, T was the trehalose treatment group, and CK was the control group.

**Figure 8:**
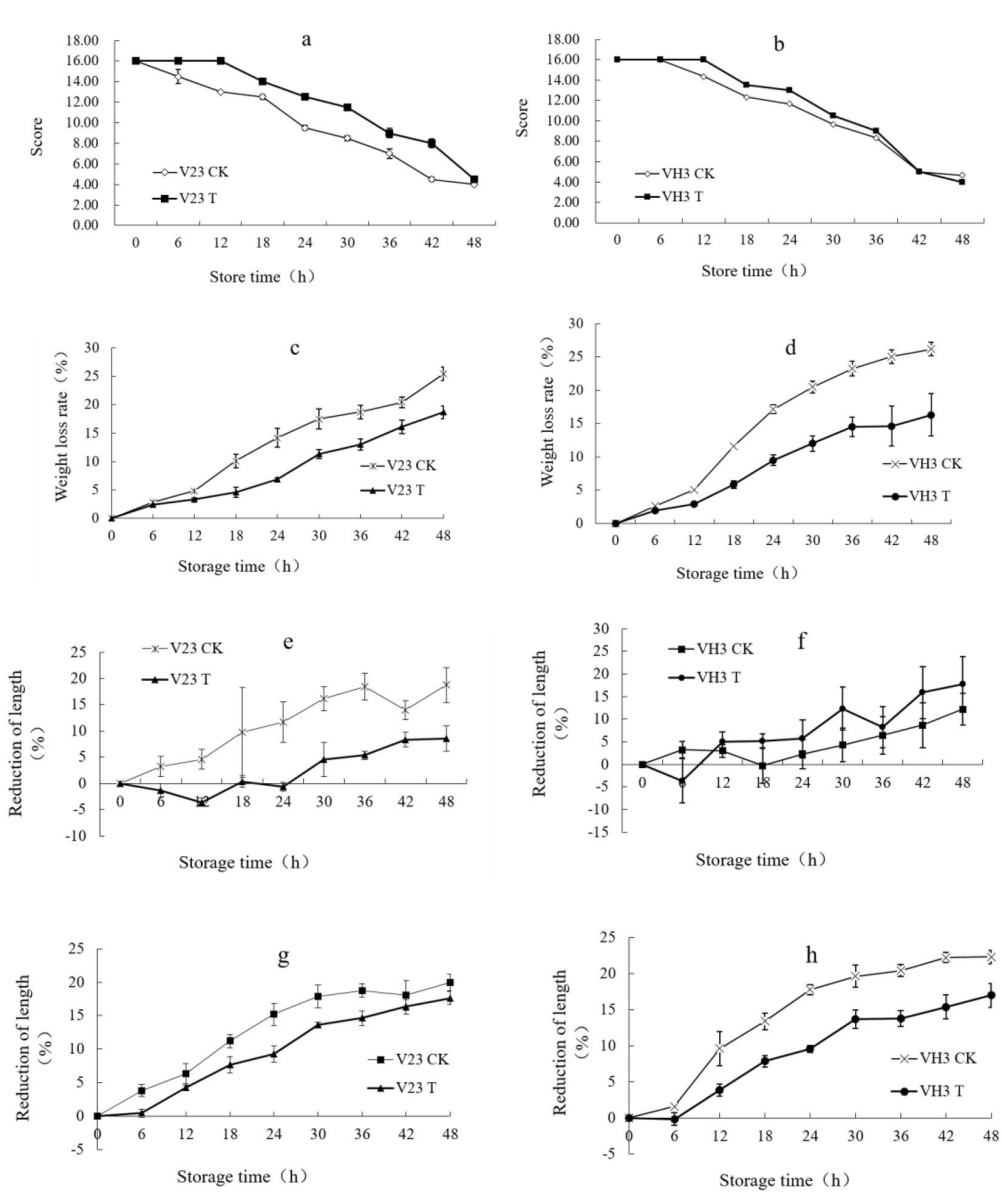
Sensory indices of the V23 and VH3 strains: trehalose treatment group (T), and blank treatment group (CK). Trend chart of sensory quality. (**a**, **b**) Weight loss rate. (**c**, **d**) Bottom diametershortening rate. (**e**, **f**) Middle diameter shortening rate (**g**, **h**). Values are means ± standard errors (n = 3). Vertical bars indicate the standard errors of the means, where the bars exceed the size of the symbol used.

The sensory quality of the *V*. *volvacea* fruit bodies under different treatment conditions at 4 °C is shown in Fig. 7. The morphological changes of strains V23 and VH3 were observed and recorded every six hours, and the evaluation indexes in Table 2 were adopted. The sensory quality score is shown in Fig. 8a,b. The sensory score of trehalose treatment group (T) were significantly higher than that of the blank control (CK) (*P* < 0.05) both in V23 and VH3, indicating that exogenous trehalose treatment had a good effect on the *V*. *volvacea* fruit bodies during storage.

#### Weight loss rate

Under 4 °C storage, the weight loss rate of the trehalose treatment group (T) and blank treatment group (CK) of V23 strain fruit bodies both showed an increasing trend (Fig. 8c). The weight loss rate of blank treatment group (CK) significantly increased at 12 h and stabilized at 30 h. The weight loss rate of trehalose treatment (T) significantly increased at 18 h and remained stable at 30 h. The weight loss rate of the blank treatment group (CK) was higher than that of the trehalose treatment group at all time points except at 6 h.

The weight loss rate of the trehalose treatment group (T) and blank treatment group (CK) of VH3 strain fruit bodies both showed an increasing trend (Fig. 8d). The weight loss rate of the blank treatment group significantly increased at 6 h and reached a stable trend at 42 h. The weight loss rate of the trehalose treatment group significantly increased at 12 h and reached a stable trend at 36 h. The weight loss rate of the blank treatment group (CK) was higher than that of the trehalose treatment group (T) at all time points except at 6 h.

#### Bottom diameter shortening rate

Under the storage at 4 °C, the shortening rate of the bottom diameter of the trehalose-treated group (T) and blank-treated group (CK) of V23 strain showed an upward trend (Fig. 8e). The blank treatment group showed a gradual increase at each time point, of which the shortening rate decreased at 42 h. The shortening rate of the trehalose-treated group after the first 18 h of storage was negative, and it increased from 30 h to 48 h of storage. The shortening rate of the blank treatment group was higher than that of trehalose treatment group at all time points.

The shortening rate of the bottom diameter of the trehalose-treated group (T) and blank-treated group (CK) of VH3 strain showed an upward trend (Fig. 8f). The blank treatment group showed a non-significant increase at each time point, of which the shortening rate decreased at 18 h. The trehalose treatment group had a negative shortening rate at the first 6 h of storage, and then increased at all time points. However, shortening rate decreased at 36 h. The shortening rate of the blank treatment group had no significant difference with the trehalose treatment group at all time points.

#### Middle diameter shortening rate

Under the storage at 4 °C, the shortening rate of the middle diameter of treatment group (T) and blank treatment group (CK) of V23 strain showed an upward trend (Fig. 8g). The blank control group was significantly increased at 6 h and reached a stable trend after 30 h. The trehalose treatment group was significantly increased at 12 h, indicating that the fruit bodies began to significantly change after 12 h. V23 strains of fruit bodies, trehalose treatment group before 36 h at various time points, middle diameter shortening rate were significantly lower than those in the blank control group. After 36 h, both sets of fruit bodies atrophied to an unacceptable state, with no significant difference.

The middle diameter shortening rate of VH3 strain fruit bodies of treatment group (T) and blank treatment group (CK) showed an upward trend (Fig. 8h). The blank control group at 12 h significantly increased and subsequently maintained a steady trend after 30 h. The trehalose treatment group also significantly increased at 12 h and after 30 h to maintain a relatively stable trend. All time points were significantly lower than the blank group except at 6 h of storage, indicating that the trehalose treatment of fruit bodies of VH3 strain has a positive effect on, reducing the middle diameter shortening rate both in V23 and VH3.

## Discussion

**Figure 9:**
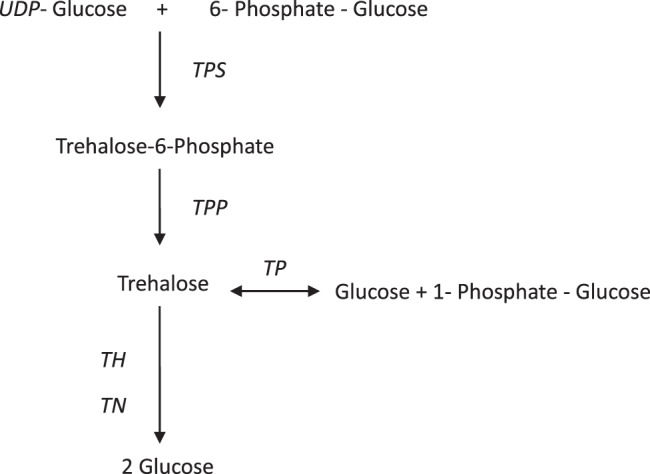
Trehalose metabolic pathway of *V*. *volvacea*.

*V*. *volvacea* is a high-temperature mushroom, therefore the mycelia growth and the development of fruit body need relatively high temperatures. Such phenomena as softening, seepage, browning and other autolysis are observed after the fruit bodies in conventional 4 °C reservations. The strong respiration of *V*. *volvacea* cells at the maturity stage and the action of polyphenol oxidase and protease lead to autolysis. However, there are no reports that clearly explain the mechanism of action of this phenomenon^19^. Therefore, we analyzed the data of the low-temperature performance of the *V*. *volvacea* and examined that the genes expression involved in trehalose-related enzymes. Trehalose exists in a variety of different metabolic pathways^20–23^. According to the amino acid sequences of enzymes related to trehalose metabolism in homologous species, such as *Coprinus cinereus* and *Laccania bicolor*, the amino acid sequences of the enzymes related to trehalose metabolism were compared in NCBI, and the gene sequences of these enzymes were screened from the genome of *V*. *volvacea*. The trehalose metabolic pathway in *V*. *volvacea*, as shown in Fig. 9, was estimated. On the one hand, the relative transcriptional expression levels of enzymes involved in the trehalose metabolic pathway were determined in mycelia of *V*. *volvacea* treated with low temperature for various periods of time. On the other hand, the sensory evaluation indices of fruit bodies stored at 4 °C after harvest were examined, which were applied by exogenous trehalose during the cultivation period.

### The relationship between the key genes of trehalose metabolic pathway and the low-temperature tolerance of *V*. *volvacea*

The mushroom V23 strain is a low-temperature sensitive strain. The low-temperature tolerance of VH3 strain is better than that of V23 strain. The two strains were different in the expression of each enzyme involved in trehalose metabolism when stimulated by hypothermia. The analysis of gene expression changes of *V*. *volvacea* mycelia under low temperature at different times showed that the metabolism of trehalose may have a postive effect on the adaptation of *V*. *volvacea* to low temperature stress.

Compared with the untreated group (0 h), the changes of various genes expression under low temperature stress in V23 and VH3 were shown in Fig. 6. During the first 2 h of low temperature stress, there was no significant difference in the expression of *TPS* gene involved in trehalose synthesis, while the expression of *TPP* gene decreased significantly in both strains. The expression of *TN* gene in V23 strain and *TH* gene in VH3 strain, involved in the degradation of trehalose, showed an upward trend in 4 h of low temperature stress. However, the intracellular trehalose was not significantly changed at 2 h of low temperature stress. Perhaps because the expression of the *TP* gene, which increased both in V23 and VH3, was more biased towards the synthesis pathway^20,24^, that is to say the TP enzyme may be a key enzyme involved in the trehalose synthesis during the early stage of exposure to low temperature stress, as reported that the *TPS/TPP* pathway plays an important role during the middle and later stages^20^. Notably, the *TPS* gene significantly increased both in V23 and VH3 at 4 h of low temperature stress. What is more, the expression of *TPS* gene in VH3 was significantly higher than that in V23. The expression of *TPP* gene was almost unchanged in VH3, but it decreased dramatically in V23. The expression of *TH* gene increased significantly in both strains, while the expression of *TN* gene increased in V23 strain and decreased in VH3 strain except for at 8 h. The expression level of *TP* gene decreased both in V23 and VH3 with time, and the expression level of it was higher in VH3 than that in V23. When *V*. *volvacea* mycelia was treated under low temperature at 4 h, there was no significant change in trehalose accumulation in VH3 strain, while the decrease of trehalose content in V23 strain was observed, which was consistent with changes of genes expression of enzymes related to trehalose metabolism. For 6 h and 8 h of low temperature stress, the expression level of *TPS* gene increased significantly (except for V23 at 8 h). The expression of *TPP* gene showed a significant decrease in V23 compared with its untreated group (0 h), and the expression level of it in VH3 was significantly higher than that in V23. The expression of *TH* gene and *TN* gene began to decrease in V23 and VH3. However, at the low temperature treatment of 8 h, the expression level of *TN* gene increased significantly in VH3 strain. The expression level of *TP* gene decreased significantly in both strains during that time, while it was higher in VH3 than that in V23. During the whole process, the expression of key genes in trehalose metabolic pathway was consistent with the changes of trehalose content in mycelia of *V*. *volvacea*. The expression of trehalose synthesis related genes (*TPS*, *TPP* and *TP*) in VH3 strain was higher than that in V23 strain at most time of low temperature stress, and the trehalose content in VH3 strain was higher than that in V23 strain, which may be one of the reasons that why VH3 strain was more tolerant to the low temperature stress than V23 strain, and it could also prove that trehalose is beneficial for fungi in response to low temperature stress.

From the recent results of the enzymatic functions of trehalose metabolism in fungi, the above results on the function of key genes in the trehalose metabolic pathway of *V*. *volvacea* were confirmed. For example, Guo Yonghao^25^ and Chen Hongge *et al*.^26^ constructed the *pUC18* and *pET-30a* vectors of the trehalose synthase gene *TPS1* from *Saccharomyces uvarum* and *Saccharomyces cerevisiae*, respectively, which were successfully expressed in Escherichia coli. Song *et al*.^27^ constructed antagonistic yeast strains overexpressing *TPP* to enhance the ability of resisting unfavorable environments. Due to the independent catalytic action of *TH* and *TN*, the fungal extracellular and cytoplasmic trehalose molecules were respectively decomposed. Therefore, the case that these two types of enzymes coexist in the organism and work together to ensure the utilization of cells for the decomposition of exogenous trehalose is typical^28^. NWAKA *et al*.^29^ found a *TH* mutant in *S*. *cerevisiae*. Trehalose utilization within the cell was not affected. However, exogenous trehalose could not grow normally as a carbon source. In contrast, *TN* gene mutants were normally grown when trehalose was contained in the medium. However, the *TH* mutant of the filamentous fungus *Neuropora crassa* did not grow on the substrate of the trehalose carbon source. This mutation effectively degrades cytoplasmic trehalose as wild-type strains^30^. FOSTER *et al*.^28^ showed that both *TN* of *Aspergillus nidulans* and *Magnaporthe grisea* can decompose intracellular trehalose. In the 1990s, to demonstrate the relationship between trehalose content and yeast tolerance, studies investigated the effect of trehalose hydrolase gene regulation on yeast cell tolerance by genetic engineering, for example, by employing gene knockout. The logarithmic growth phase strain and normal strain of the *TH*-deficient mutant, *TN*-deficient mutant and mutant strain with simultaneous deletion of *TH* and *TN* were frozen at −20 °C for 3 weeks, and the viability count and the frozen dough fermentation ability were respectively measured. The results showed that the trehalose accumulation and cell density of all mutant strains were higher than those of the parental strain. Frozen yeast dough fermentation mutant strains was higher than that in the parent, indicating that mutant strains greatly improved resistance to freezing^31^. HAN *et al*.^32^ cloned the *Pleurotus sajorcaju TP* gene. The gene in mycelium and fruit bodies of the cap and stigma were expressed and transferred the *TPS* gene and *TPP* gene double mutant to yeast cells, and these yeast cells grew in the glucose matrix. Moreover, the amount of trehalose in cells transfected with the *TP* gene was 2 to 2.5 times higher than that of the *TPS* gene, and double mutant yeast cells lacking *TPP* gene or empty vector control yeast cells. However, the catalytic activity of *TP* is reversible. Studies have reported that its catalytic orientation is more likely inclined to the trehalose synthesis process^24^, which is consistent with the results of the present study. However, the specific catalysis of trehalose in *V*. *volvacea* still needs to be further studied.

### Effect of intracellular trehalose on the low-temperature tolerance of the mycelia of *V*. *volvacea*

Trehalose can help organisms resist adverse circumstances^33^. Once the organism perceives external signals, mechanisms related to trehalose metabolism are triggered and the metabolic pathways are adjusted^34^. In this study, the observed changes in trehalose content indicated that the level of intracellular trehalose in the mycelia of *V*. *volvacea* distinctively decreased during this time period when the fungi were exposed to low temperature (0 °C). High trehalose content was found in mycelia of *V*. *volvacea* during the first 2 hours of low temperature stress. However, the expression of trehalose synthesis related genes (*TPS*/*TPP*) was not very high in the initial stage of low temperature stress. The expression of TP gene increased significantly at 2 h of low temperature stress compared to their respective blank control both in V23 and VH3, which may contribute to the high trehalose content determined during early period of low temperature stress. In terms of the change of fruit body corruption after the low temperature of *V*. *volvacea*, VH3 is more durable freshness than V23. The content of trehalose in VH3 was always higher than that of V23, which may contribute to the stronger tolerance of VH3 to low temperature stress. The trehalose content in the mycelia of *Pleurotus eryngii* var. *tuoliensis and F*. *velutipes* exposed to 37 °C heat shock increased during the early stage of processing^34,35^. So the trehalose in edible fungi may have a positive effect on tolerance to temperature stress.

### Effect of extrogenous trehalose on the low-temperature tolerance of the fruit body of *V*. *volvacea*

Several studies have suggested that exogenously added trehalose solution can enhance the tolerance of organism to temperature stress^36,37^. In the present study, for the first time, we attempted to apply exogenous trehalose solution during the cultivation of fruit bodies of *V*. *volvacea* and to explore whether the anti-hypothermia of the fruit body can be improved.

With the prolongation of storage time, the comprehensive scores of sensory qualities, including hardness, flavor, color and water effluent, showed a declining trend when the *V*. *volvacea* fruit bodies were stored at 4 °C. In the untreated blank group, the comprehensive score of sensory quality was significantly lower than that of trehalose solution-treated group at 4 °C both in V23 and VH3. In the blank control group, the comprehensive score of various sensory indices of V23 strain was lower than that of the VH3 strain. The sensory quality results showed that the addition of trehalose played a positive role on the fruit bodies under low temperature storage.

The weight loss rate of *V*. *volvacea* fruit bodies showed an upward trend with storage time prolonged at 4 °C. The weight loss rate of fruit bodies in trehalose treatment group was significantly lower than that of the blank control group. These results indicated that exogenous trehalose had a relevance to the decreased weight loss rate during the low temperature storage period of *V*. *volvacea* fruit bodies.

During the initial 24 h, the bottom diameter shortening rate of trehalose treatment was negative in V23, presumably due to the cotinued growth of fruit bodies; after 24 h, the fruit bodies further wilted. As a result, the bottom diameter shortening rate steadily increased. The bottom diameter shortening rate of the fruit bodies of VH3 strain in trehalose treatment group had no significant difference with the blank group at each time point. The bottom diameter shortening rate of V23 strain fruit bodies in the trehalose treatment group was lower than that in the blank control group, which indicated trehalose treatment had a more positive effect on the reduction of the diameter at the bottom of the fruit bodies of low temperature sensitive strain (V23) than low temperature tolerant strain (VH3).

With the 4 °C low-temperature storage time, the middle diameter shortening rate of *V*. *volvacea* fruit bodies showed an upward trend, indicating that fruit bodies shrinkage after dehydration. The middle diameter shortening rate of trehalose treated groups in V23 or VH3 strain was significantly lower than that of the respective blank control group. This finding indicated that the exogenous trehalose added had a good effect on the decrease of middle diameter shortening rate of *V*. *volvacea* fruit bodies stored at 4 °C.

In summary, during cultivation, *V*. *volvacea* was sprayed with water containing the osmolyte trehalose, which improved the tolerance of fruit bodies stored under 4 °C. Sensory traits of various indicators were slower reduced to varying degrees in the trehalose treatment group compared with the control group. The autolysis of fruit bodies under low temperatures is primarily manifested as softening, liquefaction, odor, and weight loss. Bottom diameter shortening rate measured the softening level of fruit bodies. Data analysis showed that the sensory quality of fruit bodies in the trehalose treatment group decreased to levels less than that in the blank control group. Speculated trehalose treatment of fruit bodies of *V*. *volvacea* tolerance to 4 °C has a role, consistent with the findings of HIRASAWA *et al*.^38^, that the direct addition of exogenous trehalose increased the freeze tolerance of yeast. In addition, DINIZ-MENDES *et al*.^39^ also found that treatment with exogenous trehalose, *Saccharomyces cerevisiae* also showed enhanced cell viability under freezing conditions. MERIC^40^ and SHIMA *et al*.^13^ showed that the intracellular trehalose content of 4~5% protected yeast cells from freezing injury. Thus, the addition of exogenous trehalose could improve the tolerance of *V*. *volvacea* fruit bodies to low temperature stress, especially for the low temperature sensitive strain V23.

## Conclusions

The trehalose metabolic pathway-related genes, and the content of trehalose in different treatments was determined. Furtherly, the addition of exogenous trehalose to examine the role of this sugar under the low temperature stress of *V*. *volvacea* fruit bodies revealed that trehalose could enhance the tolerance of this mushroom to low temperature stress. The intracellular trehalose content was significantly higher in VH3 strain than that in V23 strain, which may help VH3 to be more tolerant to low temperature stress. And *TP* gene may be a key gene of trehalose metabolism, which could be inclined to synthesize trehalose during low temperature stress. The application of exogenous trehalose solution during the cultivation of *V*. *volvacea* could improve the anti-hypothermia of fruit bodies stored at 4 °C, which further verified the positive correlation between trehalose and *V*. *volvacea* tolerance to low temperatures, which laid the foundation for further research on prolongation of the preservation of *V*. *volvacea* strains and fresh keeping of its fruit bodies after harvest under conventional low temperature.

## Electronic supplementary material

Supplementary Information

Dataset 1
