# Supplementary material for: Gene expression related to trehalose metabolism and its effect on Volvariella volvacea under low temperature stress
Source: Sci Rep. 2018 Jul 20;8:11011. doi: 10.1038/s41598-018-29116-z (PMC6054667; doi:10.1038/s41598-018-29116-z)
Supplement: Supplementary file 1 — Supplementary Information [file 41598_2018_29116_MOESM1_ESM.docx]

Supplementary Information

**Gene expression related to trehalose metabolism and its effect on** ***Volvariella volvacea* under low temperature stress**

Xu Zhao^1,2^, Xiaoxia Song^1,2^, Yapeng Li^1,2^, Changxia Yu^1,2^, Yan Zhao^1,2^*, Ming Gong^1,2^, Xuexiang Shen^1,2^, Mingjie Chen^1,2^*

^1^Institute of Edible Fungi, Shanghai Academy of Agricultural Sciences, Shanghai 201403, PR China

^2^National Engineering Research Center of Edible Fungi, Key Laboratory of Edible Fungi Resources and Utilization (South), Ministry of Agriculture, Shanghai 201403, PR China

* Correspondence: Yan Zhao: [jiandan289@126.com](mailto:sxx8866@163.com); Mingjie Chen: [mjchen@saas.sh.cn](mailto:mjchen@saas.sh.cn)

Tel.: +86-21-3719-6813 fax: +86-21-6220-1337

**Table S1.** The Genomic DNA elimination reaction system

| Reagent | Volume |
| --- | --- |
| 5×gDNA Erase Buffer | 2 μL |
| gDNA Eraser | 1 μL |
| Total RNA | 1 μg |
| RNase Free dH_2_O | up to 10 μL |

In SYBR® Green qPCR assay 20 μL reaction system, adjust the total RNA content at 1 μg，after the total RNA concentration was detected, the volume was added. The reaction conditions: 42 °C，2 min（or room temperature 5 min）, 4 °C save.

**Table S2.** Reverse transcription reaction system

| Reagent | Volume |
| --- | --- |
| 5×PrimeScript Buffer 2 （for Real Time） | 4 μL |
| PrimeScript RT Enzyme Mix I | 1 μL |
| RT Primer Mix | 1 μL |
| Reaction Solution from Step 1 | 10 μL |
| RNase Free dH_2_O | up to 20 μL |

The reaction conditions: 37 °C, 15 min；85 °C, 5 sec; 4 °C keep. cDNA can keep in﹣20 °C.

**Table S3.** Reaction system of PCR

| Reagent | Volume |
| --- | --- |
| Taq Enzyme Buffer(10×) | 2.5 μL |
| dNTP(10mmol/L) | 1 μL |
| Forward Primer | 1 μL |
| Reverse Primer | 1 μL |
| Template DNA | 1 μL |
| Tap Enzyme (5 U/uL) | 0.25 μL |
| ddH_2_O | 18.25 μL |
| Total | 25 μL |

The reaction conditions were 94 °C, 3 min followed by 35 cycles at 94 °C for 30s,

55 °C for 30 s, and 72 °C, for 30 s, with a final extension at 72 °C for 10 min. Agarose gel electrophoresis was performed on a 1.5% agarose gel to test the amplified products.

**Table S4.** The reaction system of ligation

| Reagent | Volume |
| --- | --- |
| 2×Rapid Ligation Buffer | 5 μL |
| pGEM**^®^**-T Vector | 0.5 μL |
| PCR Product | 2 μL |
| T4 DNA Ligase | 1 μL |
| ddH_2_O | 1.5 μL |
| Total | 10 μL |

**Table S5.** Parameters of standard curve

| Gene | Slope | R^2^ | Amplification Efficiency |
| --- | --- | --- | --- |
| *TUB* | -3.066 | 1.000 | 111.913 |
| *TPP* | -2.982 | 0.990 | 116.427 |
| *TPS* | -2.843 | 0.997 | 124.746 |
| *TH* | -3.210 | 0.990 | 104.893 |
| *TN* | -3.185 | 0.999 | 106.064 |
| *TP* | -3.270 | 1.000 | 102.209 |

According to the standard curve of the reference gene and target genes, the CT values between target gene and reference gene of the corresponding points of plasmid concentration were respectively made subtraction, and then five ΔCT value of dilution points were obtained. In the Excel table, the corresponding difference values of five points are plotted. The slope K met︱ΔK︱≤0.1, so the method of ΔΔCT was used to do the relative quantification of target genes.

**Table S6.** The slope of ΔCT value

| Target Gene | The slope of ΔCT value (K value) |
| --- | --- |
| *TPP* | 0.0279 |
| *TPS* | 0.0742 |
| *TH* | -0.0420 |
| *TN* | -0.0396 |
| *TP* | -0.0681 |

**Table S7.** Expression of *TPP* gene at low temperature in V23

| Processing time under 0 °C（h） | Average CT value ­of *TPP* | - Average CT value of *TUB* | Relative Content |
| --- | --- | --- | --- |
| 0 | 22.27 | 20.35 | 1.00 |
| 2 | 22.12 | 19.12 | 0.47 |
| 4 | 23.56 | 19.97 | 0.31 |
| 6 | 22.15 | 19.72 | 0.70 |
| 8 | 24.01 | 21.34 | 0.59 |

Expression of *TPP* gene at low temperature in VH3

| Processing time under 0 °C（h） | Average CT value ­of *TPP* | - Average CT value _­_of *TUB* | Relative Content |
| --- | --- | --- | --- |
| 0 | 23.33 | 18.57 | 1.00 |
| 2 | 25.03 | 18.12 | 0.23 |
| 4 | 22.19 | 17.44 | 1.01 |
| 6 | 23.48 | 18.58 | 0.91 |
| 8 | 23.85 | 18.79 | 0.81 |

**Table S8.** Expression of *TPS* gene at low temperature in V23

| Processing time under 0 °C（h） | Average CT value ­of *TPS* | - Average CT value _­_of *TUB* | Relative Content |
| --- | --- | --- | --- |
| 0 | 23.82 | 21.07 | 1.00 |
| 2 | 25.09 | 22.30 | 0.96 |
| 4 | 24.78 | 22.50 | 1.38 |
| 6 | 23.51 | 21.51 | 1.67 |
| 8 | 26.95 | 23.68 | 0.69 |

Expression of *TPS* gene at low temperature in VH3

| Processing time under 0 °C（h） | Average CT value of *TPS* | - Average CT value _­_of *TUB* | Relative Content |
| --- | --- | --- | --- |
| 0 | 23.88 | 21.31 | 1.00 |
| 2 | 24.53 | 21.76 | 0.87 |
| 4 | 22.77 | 20.91 | 1.65 |
| 6 | 22.90 | 20.95 | 1.53 |
| 8 | 24.91 | 22.70 | 1.28 |

**Table S9.** Expression of *TH* gene at low temperature in V23

| Processing time under 0 °C（h） | Average CT value ­of *TH* | - Average CT value _­_of *TUB* | Relative Content |
| --- | --- | --- | --- |
| 0 | 24.22 | 22.55 | 1.00 |
| 2 | 23.45 | 21.94 | 1.11 |
| 4 | 24.61 | 23.37 | 1.34 |
| 6 | 25.21 | 23.16 | 0.76 |
| 8 | 25.93 | 23.02 | 0.42 |

Expression of *TH* gene at low temperature in VH3

| Processing time under 0 °C（h） | Average CT value ­of *TH* | - Average CT value _­_of *TUB* | Relative Content |
| --- | --- | --- | --- |
| 0 | 26.32 | 24.07 | 1.00 |
| 2 | 22.71 | 21.18 | 1.65 |
| 4 | 23.05 | 21.35 | 1.47 |
| 6 | 24.72 | 21.76 | 0.61 |
| 8 | 24.64 | 22.08 | 0.81 |

**Table S10.** Expression of *TN* gene at low temperature in V23

| Processing time under 0 °C（h） | Average CT value ­of *TN* | - Average CT value _­_of *TUB* | Relative Content |
| --- | --- | --- | --- |
| 0 | 29.56 | 23.79 | 1.00 |
| 2 | 28.60 | 23.01 | 1.13 |
| 4 | 29.91 | 24.51 | 1.29 |
| 6 | 31.83 | 24.38 | 0.31 |
| 8 | 32.09 | 24.38 | 0.26 |

Expression of *TN* gene at low temperature in VH3

| Processing time under 0 °C（h） | Average CT value of *TN* | - Average CT value _­_of *TUB* | Relative Content |
| --- | --- | --- | --- |
| 0 | 30.89 | 25.33 | 1.00 |
| 2 | 28.44 | 22.71 | 0.89 |
| 4 | 29.23 | 23.09 | 0.67 |
| 6 | 30.00 | 23.40 | 0.48 |
| 8 | 29.33 | 24.04 | 1.20 |

**Table S11.** Expression of *TP* gene at low temperature in V23

| Processing time under 0 °C（h） | Average CT value ­of *TP* | - Average CT value _­_of *TUB* | Relative Content |
| --- | --- | --- | --- |
| 0 | 20.23 | 22.58 | 1.00 |
| 2 | 20.34 | 22.20 | 1.14 |
| 4 | 20.81 | 21.76 | 0.35 |
| 6 | 20.80 | 22 | 0.42 |
| 8 | 19.97 | 20.24 | 0.20 |

Expression of *TP* gene at low temperature in VH3

| Processing time under 0 °C（h） | Average CT value ­of *TP* | - Average CT value _­_of *TUB* | Relative Content |
| --- | --- | --- | --- |
| 0 | 14.05 | 17.72 | 1.00 |
| 2 | 20.02 | 21.89 | 1.29 |
| 4 | 15.60 | 18.67 | 0.70 |
| 6 | 16.30 | 19.23 | 0.65 |
| 8 | 18.18 | 21.27 | 0.85 |

**Original figures**

**Figure S1**

| Figure after editing by cropping, labelling the lines and splitting color channels. 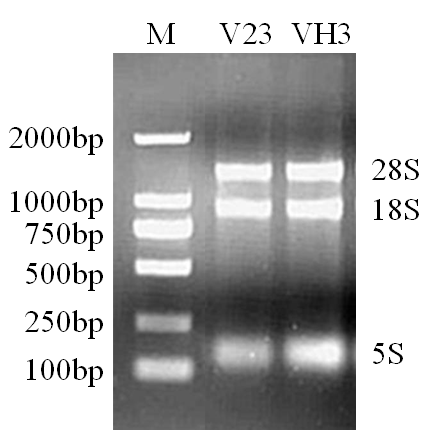 | Figure before editing  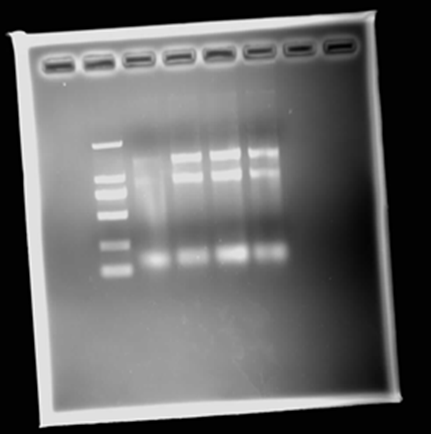  M V23 VH3 |
| --- | --- |
